# Effect of physical exercise on spontaneous physical activity energy expenditure and energy intake in overweight adults (the EFECT study): a study protocol for a randomized controlled trial

**DOI:** 10.1186/s13063-018-2445-6

**Published:** 2018-03-07

**Authors:** Vitor Barreto Paravidino, Mauro Felippe Felix Mediano, Inácio Crochemore M Silva, Andrea Wendt, Fabrício Boscolo Del Vecchio, Fabiana Alves Neves, Bruno de Souza Terra, Erika Alvarenga Corrêa Gomes, Anibal Sanchez Moura, Rosely Sichieri

**Affiliations:** 1 Department of Epidemiology, Institute of Social Medicine, State University of Rio de Janeiro, Rio de Janeiro, Brazil; 2 Department of Physical Education and Sports, Naval Academy – Brazilian Navy, Rio de Janeiro, Rio de Janeiro, Brazil; 3 Evandro Chagas National Institute of Infectious Disease, Oswaldo Cruz Foundation, Rio de Janeiro, Rio de Janeiro, Brazil; 4 Post-graduate Program in Epidemiology, Federal University of Pelotas, Pelotas, Brazil; 5 Post-Graduate Program in Physical Education, Federal University of Pelotas, Pelotas, Rio Grande do Sul Brazil; 6 Department of Physiology Science, State University of Rio de Janeiro, Rio de Janeiro, Brazil; 7 Research Laboratory of Exercise Sciences, Physical Education Center Admiral Adalberto Nunes, Brazilian Navy, Rio de Janeiro, Rio de Janeiro, Brazil; 8 Laboratory of Exercise Sciences, Fluminense Federal University, Niterói, Rio de Janeiro Brazil

**Keywords:** Obesity, Exercise training, Physical activity energy expenditure, Energy intake, Compensatory effect

## Abstract

**Background:**

Physical exercise interventions have been extensively advocated for the treatment of obesity; however, clinical trials evaluating the effectiveness of exercise interventions on weight control show controversial results. Compensatory mechanisms through a decrease in energy expenditure and/or an increase in caloric consumption is a possible explanation. Several physiological mechanisms involved in the energy balance could explain compensatory mechanisms, but the influences of physical exercise on these adjustments are still unclear. Therefore, the present trial aims to evaluate the effects of exercise on non-exercise physical activity energy expenditure, energy intake and appetite sensations among active overweight/obese adults, as well as, to investigate hormonal changes associated with physical exercise.

**Methods:**

This study is a randomized controlled trial with parallel, three-group experimental arms. Eighty-one overweight/obese adults will be randomly allocated (1:1:1 ratio) to a vigorous exercise group, moderate exercise group or control group. The trial will be conducted at a military institution and the intervention groups will be submitted to exercise sessions in the evening, three times a week for 65 min, during a 2-week period. The primary outcome will be total spontaneous physical activity energy expenditure during a 2-week period. Secondary outcomes will be caloric intake, appetite sensations and laboratorial biomarkers. Intention-to-treat analysis will be performed using linear mixed-effects models to evaluate the effect of treatment-by-time interaction on primary and secondary outcomes. Data analysis will be performed using SAS 9.3 and statistical significance will be set at *p* < 0.05.

**Discussion:**

The results of the present study will help to understand the effect of physical exercise training on subsequent non-exercise physical activity, appetite and energy intake as well as understand the physiological mechanisms underlying a possible compensatory phenomenon, supporting the development of more effective interventions for prevention and treatment of obesity.

**Trial registration:**

Physical Exercise and Energy Balance trial registry, trial registration number: NCT 03138187. Registered on 30 April 2017.

**Electronic supplementary material:**

The online version of this article (10.1186/s13063-018-2445-6) contains supplementary material, which is available to authorized users.

## Background

Obesity is an important multifactorial condition associated with an increased risk of many chronic diseases and high mortality rates [1]. The prevalence of obesity has been increasing over the last decades, mainly due to an imbalance between energy intake and expenditure during an extended time period [2, 3].

Physical exercise interventions have been extensively advocated for the treatment of obesity [2]. The rationale is to facilitate weight loss, generating a negative energy balance by increasing total energy expenditure. However, some clinical trials evaluating the effectiveness of exercise interventions on weight control did not confirm this hypothesis [4–6].

The small or even null effect of exercise on weight management observed in some studies could be explained by the lower energy deficit actually induced by exercise training in comparison to those theoretically predicted. According to the activitystat hypothesis [7], an increase in physical activity levels in one moment is compensated by decreasing physical activities in another moment in order to maintain an overall physical activity set point [8, 9]. This compensatory energy expenditure effect was observed by some [10, 11] but not confirmed by others authors [12].

Another important aspect that could wane the influence of exercise on weight loss is the potential increase in food consumption as a compensatory effect of an exercise-induced energy expenditure [13]. Some studies have demonstrated that exercise training can increase hunger and promote overfeeding, decreasing the expected negative energy balance and making weight loss more difficult [14, 15]. However, other studies showed that appetite and caloric intake are decreased or unchanged after physical activities [16, 17]. The conflicting results observed in the academic literature may be related to the heterogeneity of the studies, in which large methodological differences (study population, exercise protocols, evaluated variables) are observed.

Several physiological mechanisms, particularly hormonal, are involved in the energy expenditure and intake regulation and can explain these behavioral compensatory mechanisms [18, 19]. However, the existing interactions and the influence of physical exercise on these adjustments are still unclear. Therefore, it is necessary to better understand the relationship between physical exercise and energy expenditure, as well as the possible interdependence between energy expenditure and food consumption and the hormonal regulatory mechanisms related to energy balance in overweight individuals. The influence of different exercise intensities on energy expenditure and food intake should also be investigated. The EFECT study, which stands for “Physical Exercise and Compensatory Effects” in Portuguese, is designed to address this gap in the academic literature. For the main outcome, we hypothesize that a greater reduction on spontaneous physical activity energy expenditure will be observed in the vigorous exercise group (VEG) after the 15-day follow-up.

## Objectives

### Primary objective

1. To evaluate the effect of different exercise intensities on spontaneous physical activity energy expenditure and energy intake

### Secondary objectives

1. To evaluate hunger/satiety associated with exercise intensity
2. To investigate hormonal changes associated with exercise intensity

## Methods

### Study design

**Fig. 1:**
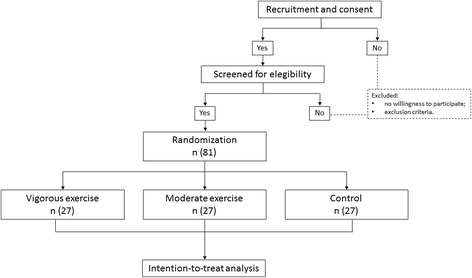
Planned flow diagram

**Fig. 2:**
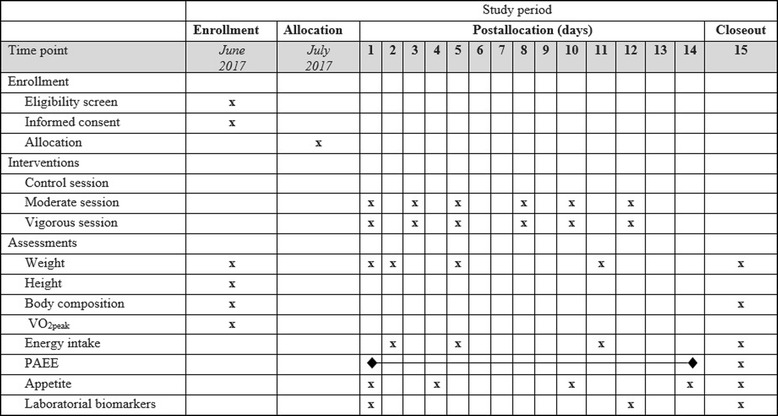
SPIRIT diagram

A randomized controlled trial, designed to evaluate the effect of structured physical exercise sessions on the spontaneous physical activity energy expenditure and caloric intake in overweight adults. The design employs parallel, three-group experimental arms: (1) a moderate exercise group (MEG); (2) a vigorous exercise group (VEG); and a control group (CG) without physical exercise sessions. The planned flow diagram of this trial is presented in Fig. 1. The protocol is reported according to the Standard Protocol Items: Recommendations for Interventional Trials (Fig. 2 SPIRIT diagram and Additional file 1: SPIRIT checklist).

### Study setting

The trial will be conducted at the Naval Academy (Brazilian Navy, Rio de Janeiro, Brazil) during the school year of 2017. This military academic institution allows a very controlled experimental condition to conduct this study. In this institution, approximately 900 students, aged 18 to 24 years, undergo a military training during a period of 4 years. The institution works on boarding from Monday to Friday and during the weekend students are free to go home.

During the weekdays, all students have the same routine at school: they wake up at 6 a.m., attend academic classes in the morning and physical exercise classes in the afternoon. The school offers three basic meals: breakfast at 6 a.m., lunch at 12 p.m. and dinner at 7 p.m. At 10 p.m. students must go to their bedrooms to sleep.

### Participants and eligibility criteria

Male students from the first, second, third and fourth grades will be invited to participate in the study. Those with overweight or obesity, defined as a measured Body Mass Index (BMI) equal to or greater than 25 kg∙m^-2^ [20] and a percentage of body fat (%BF) equal to or greater than 21%, evaluated by bioelectrical impedance [21] will be enrolled. Exclusion criteria are: (1) reported diabetes mellitus or cardiovascular diseases and (2) musculoskeletal injuries or any other factor that precludes the achievement of the exercise protocol.

### Intervention and exercise protocol

For students willing to participate in the study, an exercise treadmill test will be performed prior to the intervention sessions in order to evaluate clinical symptoms and physiological responses during exercise.

The intervention groups (MEG and VEG) will be submitted to exercise sessions in the evening (5–7 p.m.), performed three times a week (Monday, Wednesday and Friday) for 65 min, during a 2-week period. Exercise intensity will be set according to the oxygen uptake reserve (VO_2_R) concept, calculated considering the peak oxygen consumption directly measured during an incremental treadmill test.

Physical exercise sessions will be divided into three phases: warm-up (5 min), training (55 min) and cool-down (5 min). During warm-up and cool-down phases, for both the MEG and the VEG, participants will be instructed to walk at a low intensity.

The training phase of the MEG will consist of four sets of 10 min of walking/running at moderate intensity (40 to 59% VO_2_R), with 5 min walking at low intensity (30 to 39% VO_2_R) for recovery between sets [22]. The training phase of the vigorous exercise group (VEG) will consist of four sets of 10 min of running at vigorous intensity (60 to 89% VO_2_R), with 5 min of walking at low intensity (30 to 39% VO_2_R) for recovery between sets [22].

Participants will self-monitor their target heart rate using a heart-rate monitor Polar FT1 (Polar Electro Oy, Kempele, Finland). In addition, the researchers will monitor all physical exercise sessions to ensure that they will perform the experimental protocol properly.

Participants in the CG will not be submitted to any specific physical training protocol. No specific counseling to change the daily routine of participants during the 15 days will be provided.

### Sample size

Sample size was calculated based on a mean difference in daily energy expenditure between groups of 110 kcal [23], with a coefficient of variation equal to 1 (standard deviation of 110 kcal) [24]. The sample size required for the study, with an *α* of 0.05 (two-sided), *β* of 0.10 and estimating a drop out of 20%, is 81 adolescents (i.e., 27 in each group) [25].

### Randomization and allocation concealment

Eighty-one participants will be randomly allocated into three groups in a 1:1:1 ratio. The randomization list will be generated in blocks and the allocation sequence will be concealed using opaque envelopes by a researcher not involved in recruitment. Block size will be blinded from investigators involved in patients’ recruitment.

### Blinding

Participants and physical trainer blinding will not be feasible due to the characteristics of prescribed exercise sessions. However, the evaluators will be blinded to the following endpoints: weight, body composition, appetite, energy intake and laboratorial biomarkers.

### Outcomes

The primary outcome will be total physical activity energy expenditure during the 2-week period. Secondary outcomes will be caloric intake, hungry and satiety sensations and biomarkers (lactate, cortisol, insulin, leptin, acylated ghrelin).

### Measurements

#### Anthropometric

Body weight will be measured using a portable electronic scale (Tanita BC-558 Japan) with a 150-kg capacity and 50-g precision, with minimal clothing and without shoes. Height will be measured using a portable stadiometer (Alturexata, Brazil) with an amplitude of 200 cm and variation of 0.1 cm. Body Mass Index will be determined as a ratio between body weight (kg) and squared height (m).

#### Body composition

Body composition will be estimated by tetrapolar bioimpedance analysis (BIA), model RJL System, according to the standard tetrapolar technique [26–28]. The electrodes will be placed at the dominant wrist and ankle. Fat-free mass (FFM) and body fat (BF) will be calculated from impedance (resistance and reactance) and anthropometry (body weight and height) data, according to RJL software parameters.

In order to obtain accurate measurements, a standardized procedure will be followed: (1) light clothes, without shoes or socks; (2) supine position for 5–10 min before measurements; (3) no eating or drinking within 4 h of the test; (4) no alcohol consumption within 12 h of the test; (5) no exercise within 8 h of the test; and (6) urinate within 30 min of the test [29].

#### Exercise treadmill testing

In order to evaluate clinical symptoms and physiological responses during exercise, a blinded exercise physiologist will perform a standard ramp protocol on a treadmill, Master Super ATL model (Inbramed, Porto Alegre, RS, Brazil).

The test will start at 8 km∙h^-1^ with 1% inclination and will continue with an increase ratio of 0.8 km∙h^-1^∙min^-1^ until exhaustion/interruption request or the observation of some criterion indicating the interruption. Ventilatory variables, blood gases and heart rate (HR) will be monitored and recorded through a MedGraphics VO2000® (Medical Graphics Corporation, Saint Paul, MN, USA) metabolic analyzer, connected to a Polar® heart-rate monitor (Polar Electro Oy, Kempele, Finland). The maximum oxygen consumption or peak oxygen consumption (VO_2_max or VO_2_peak), maximum heart rate (HRmax), total test time, perceived exertion rate (Borg scale from 0 to 10) [30] and the reason for the test interruption will be obtained from the test. In addition, through visual inspection analysis, by consensus of two experienced evaluators, ventilatory thresholds 1 and 2 will be identified. For these thresholds, the oxygen consumption in which they are observed and the HR in these stages will be recorded for prescription decisions.

The test will be interrupted according to the following criteria: fall in the variables minute ventilation (VE), oxygen consumption (VO_2_) or oxygen pulse (VO_2_∙beat^-1^) with increased load, claudication, severe headache, chest pain or patient’s inability to continue the test. The test will be considered maximum if at least one of these criteria is achieved: plateau in VO_2_, percentage of HRmax (% HRmax) predicted for age (220 − age) close to or greater than 100%, respiratory quotient (CO_2_/O_2_) greater than 1.15, rate of perceived exertion close to 10 and observation of the two ventilatory thresholds.

#### Physical activity energy expenditure

Physical activity energy expenditure will be assessed for all groups (control, MEG and VEG) by using triaxial accelerometers (ActiGraph GT3x-BT, Pensacola, FL, USA) [31]. The device will be positioned at the anterior axillary line of the non-dominant hip, for 15 consecutive days. Participants will be instructed to not remove the device during this period, except for bathing and water-based activities.

Accelerometers will be initialized to collect data in 30-Hz time resolution and in 5-s epochs. Non-wear time will be defined as ≥ 60 min of zero counts, with a minimum of 10 h constituting a valid day. Therefore, individuals will have days excluded from the analysis if they failed to provide a minimum of 600 min (10 h) of valid data.

The recorded data from the accelerometer in form of counts will be analyzed using the ActiLife 6 software (ActiGraph Manufacturing Technology Inc., FL, USA). Physical activity energy expenditure will be calculate based on equations proposed by Santos-Lozano et al. [32], which presented for adults a coefficient of determination (R^2^) of 0.71 (standard error of the estimation of 1.21 and root mean sum of squared errors of 1.20).

#### Food consumption

Food and beverage consumption will be assessed by 24-h food recalls (REC24h). Nutritionists will conduct a face-to-face interview in four different moments during the intervention period: on the second and fifth days (Tuesday and Friday) in the first week; and at the 11th and 15th days (Thursday and Monday) in the second week. These days were selected to encompass weekdays (second, fifth, 11th) and weekend (15th), and days with (second, 11th) and without (fifth, 15th) exercise session.

During the interviews, each participant will be asked to report all food and beverages consumed (except water) on the previous day, including a description of food items, the amount consumed and the time of consumption. All items at each meal will be reviewed to ensure that no item is omitted, according to the menu at the restaurant. The 24-h recalls will be analyzed using the BrasilNutri software that encompasses a computerized food database developed for the 2008–2009 nationwide dietary survey [33].

#### Subjective hunger and satiety sensations

**Fig. 3:**
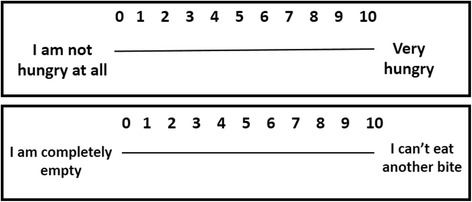
Visual analog scale of hunger and satiety sensations

Subjective hunger and satiety sensations will be measured through the Visual Analog Scale (VAS) proposed by Flint et al. [34]. The VAS is composed of lines with a length of 100 mm, where the extremities express the minimum and the maximum of the individual’s perception of hunger and satiety, separately (Fig. 3). Subjects will be asked to make a mark on this line. The marked point corresponds to their feelings about hunger or satiety. This will be measured by the definition of the individual score [34]. The evaluation will be performed before and after the exercise sessions, as well as after the main meals (breakfast, lunch and dinner) on the days of food consumption assessments.

Prior to the first exercise session, an ad-libitum buffet will be provided to all experimental groups (vigorous, moderate and control).

Participants will be asked to record hunger and satiety sensations in a diary. In order to improve adherence, a text message will be sent to the participants reminding them to complete their diary.

#### Biochemical and hormonal analyses

Blood and salivary samples will be collected to investigate physiological mechanisms involved in energy expenditure (lactate and cortisol) and food consumption regulation (insulin, leptin and acylated ghrelin), at the first (baseline) and the 15th days of the intervention period, in the morning and after at least 10 h of fasting. Also, salivary cortisol and lactate will be measured after the first and last exercise sessions.

In order to measure salivary cortisol, fasting saliva sample will be collected about an hour after waking up and stored at 4 °C in the Salivette® tube, before eating and brushing the teeth. Participants will bring the salivary sample to the researchers in the same day. The Salivette® tubes will be centrifuged at 1000 × g for 3 min at room temperature and then stored at − 20 °C for later measures, by a commercial ELISA kit. To avoid any confounding factors, the participants will be advised to not do exercise and drink alcohol in the last 24 h, rinse their mouths with water through light mouthwashes immediately prior to collection.

Blood samples will be collected into a 6-ml EDTA K3 vacuum tube with Pefabloc®, 1 mg/mL of blood. The samples will be centrifuged at 3000 × g for 15 min at 4 °C. The serum samples will be aliquoted to identify Eppendorfs for future measurements of insulin, leptin and ghrelin, and stored at – 70 °C. The ghrelin aliquot will be acidified with HCl, to a final concentration of 0.05 N before storage.

Fasting glucose and lactate will be measured using a glucometer (Accu-Chek Active, Roche Diagnostics®, Mannheim, Germany) and a lactate analyzer (Lactate Plus, Nova Biomedical Corporation®, Waltham, MA, USA), respectively. Also, fasting insulin and leptin will be evaluated by a commercial RIA kit. On the other hand, measurements of ghrelin will be performed using a commercial ELISA kit.

The value of the Homeostasis Model Assessment of Insulin Resistance (HOMA-IR) index will be calculated by using the formula:(Fasting insulin (*μ*UI ⋅ mL) × fasting glucose (mmol ⋅ L^− 1^))/22.5 [35]

### Data analysis

Descriptive analysis for variables of interest consist of means and standard deviation for continuous variables and percentage for categorical variables.

Energy expenditure associated with physical activity will be assessed in the first hour of accelerometer usage, corresponding to the period of the exercise sessions, and over the course of 15 days. Differences between sessions for energy expenditure in the first hour will be performed by using a one-way analysis of variance (ANOVA), followed by a post-hoc Scheffé test. Differences in daily and accumulated physical activity energy expenditure between groups will be performed using linear mixed models that take into account the correlations between repeated measures over time and dropouts. The models will assess the rate of change of the outcomes by the treatment-by-time interaction variable. The covariance structure of the models will be tested and residuals analyses will be performed. To deal with the potential weartime differences between groups, sensitivity analysis will be performed and a “non-wear time” variable will be created and included in the model. Data analysis will be performed using SAS 9.3 (SAS Institute Inc, Cary, NC, USA). Statistical significance will be set at *p* < 0.05 for all analyses.

## Monitoring

All adverse events that occur during the trial will be recorded and an independent committee will be consulted in case of severe adverse events to decide about the participants’ continuation in the trial.

## Discussion

Although physical exercise training has been widely recommended for the prevention and treatment of obesity [2], studies have demonstrated it is not an effective method of weight reduction [36, 37], probably due to compensatory changes, by reducing following non-exercise physical activity [10, 38] and/or increasing the energy intake [39]. However, other studies do not support this hypothesis and demonstrate that physical exercise, when prescribed and completed at a sufficient magnitude, is an effective method of weight reduction [40, 41]. Despite a large inter-individual variability in exercise-induced weight loss [42], people tend to lose less weight than is theoretically predicted [37].

A recent study in overweight adults showed that the effect of exercise training on the behavioral adaptive response was dose-dependent. Reduction of subsequent non-exercise physical activity occurred only in the group performing a high dose of exercise [43]. In addition, high-intensity exercise may change appetite-regulating hormones, leading to a greater suppression of orexigenic signals and greater stimulation of anorexigenic signals, contributing to a reduction in post-exercise energy intake, an effect known as ‘exercise-induced anorexia’ [18]. However, findings are not consistent and some other studies presented different results [44–47].

Therefore, the present study was designed to fill in this gap in the academic literature, investigating the effect of intensity of physical exercise training on subsequent non-exercise physical activity, appetite and energy intake as well as measuring possible physiological mechanisms underlying such compensatory phenomena. Highly controlled conditions for exercise and food intake in a free-living population is a hard task, which will be circumvented by studying a young military population living in the school. The results will help to understand this complex phenomenon and support the development of more effective interventions for the prevention and treatment of obesity.

This study protocol has some limitations: (1) although physical activity energy expenditure measurement is in accordance with others studies, bathing and other water-based activities will not be measured and this period will be considered as non-wear time; (2) within the first hour of waking up, cortisol increases and decreases dramatically and we may miss important changes in diurnal cortisol responses by only assessing salivary cortisol at one time point in the morning. However, as previously described, participants wake up at the same time and they will be instructed to collect the salivary sample at the same moment. In addition, the evaluation of changes in diurnal cortisol responses is beyond of the scope of the present study; and (3) the present study will use only overweight military men, making it difficult to extrapolate our results to other populations. Notwithstanding, we are investigating a complex phenomenon and a homogeneous sample seems to be more reasonable to answer our research question.

## Trial status

Participants are currently being recruited.

## Additional file

Additional file 1: SPIRIT checklist. (DOC 121 kb)

## Abbreviations

% HRmax: Percentage of maximum heart rate
%BF: Percentage of body fat
BF: Body fat
BIA: Bioimpedance analysis
BMI: Body Mass Index
CG: Control group
CO_2_/O_2_: Respiratory quotient
EFECT study: Physical exercise and compensatory effects
FFM: Fat-free mass
HOMA-IR: Homeostasis Model Assessment of Insulin Resistance
HR: Heart rate
HRmax: Maximum heart rate
MEG: Moderate exercise group
REC24h: 24-h food recalls
VAS: Visual Analog Scale
VE: Minute ventilation
VEG: Vigorous exercise group
VO_2_: Oxygen consumption
VO_2_∙beat^-1^: Oxygen pulse
VO_2_max: Maximum oxygen consumption
VO_2_peak: Peak oxygen consumption
VO_2_R: Oxygen uptake reserve
